# Resrip: a network dedicated to children and adolescents suffering from chronic rheumatic diseases in the Ile-de-France region

**DOI:** 10.1186/1546-0096-12-S1-P189

**Published:** 2014-09-17

**Authors:** Linda Rossi-Semerano, Chrystelle Hascoët, Isabelle Koné-Paut

**Affiliations:** 1 Department of Paediatrics and Paediatric Rheumatology, National Reference Center for Auto-inflammatory Diseases, Bicêtre Hospital, Le Kremlin Bicêtre, France

## Introduction

Introduction: chronic rheumatic diseases affect around 2000 children in the Ile-de-France region. Most patients suffer from juvenile idiopathic arthritis (JIA) that leads to chronic pain, fatigue and structural damage responsible for short- and long-term disability. Several physicians and health professionals may be involved in the treatment and care of these patients. This multidisciplinary approach is particularly important in managing paediatric rheumatic diseases, since many symptoms are chronic and change in severity over time. The RESRIP (Réseau pour les Rhumatismes Inflammatoires Pédiatriques) network has been created to improve and personalize patient care coordination.

## Objectives

Objectives: improvement of patients care in a non-hospital setting, development of therapeutic education at home in order to ameliorate treatment adherence; supporting the transition period from paediatric to adult health care; reduction of costs thanks to lower need for hospitalization and medical transportation.

## Methods

Patients and methods: any patient aged equal or less than 18 years suffering from chronic inflammatory diseases having given informed consent could be enrolled in the network. Patients needed to meet at least two of the following criteria: disease complexity (impact on: physical or psychological status, school attendance, growth and/or development; complexity of treatment); association of two or more diseases; necessity of at least 3 different health professionals; social difficulties; demand of support expressed by the patient or the parents; transition to adult health care; patient followed in a peripheral hospital. Physician network coordinator evaluates patient needs, establishes care process and weaves relationships between the different health professionals. A patient notebook helps this process. Health professionals enter into the network after signing the specific informed consent too. Patients and health professionals inclusions started in September 2013.

## Results

Results: at present 46 patients (29F, 17M, median age 10,8 years, median disease duration 3,1 years) have been included. Principal diseases were: JIA (32 patients: polyarticular 14, oligoarticular 10, psoriatic arthritis 3, enthesitis-related arthritis 3, systemic 2); connective tissue diseases (6), auto-inflammatory diseases 4, idiopathic uveitis 2, other 2. Seventy-five health professionals have adhered to the network.

## Conclusion

Conclusion: RESRIP is the first French network dedicated to children and adolescents with chronic rheumatic diseases, which might improve patient care coordination and treatment adherence.

## Disclosure of interest

None declared.

